# Differentiation of the Contribution of Familiarity and Recollection to the Old/New Effects in Associative Recognition: Insight from Semantic Relation

**DOI:** 10.3390/brainsci13040553

**Published:** 2023-03-25

**Authors:** Aiqing Nie, Yuanying Wu

**Affiliations:** 1 Department of Psychology, College of Educational Sciences, Shanxi Normal University, 339 Taiyu Road, Taiyuan 030031, China; 2 The MOE Frontier Science Center for Brain Science & Brain-Machine Integration, Zhejiang University, Hangzhou 310058, China; 3 Department of Psychology and Behavioral Sciences, Zhejiang University, Hangzhou 310028, China

**Keywords:** associative memory, semantic relation, familiarity, FN400, LPC, pair

## Abstract

Previous research has revealed two different old/new effects, the early mid-frontal old/new effect (a.k.a., FN400) and the late parietal old/new effect (a.k.a., LPC), which relate to familiarity and recollection processes, respectively. Although associative recognition is thought to be more based on recollection, recent studies have confirmed that familiarity can make a great contribution when the items of a pair are unitized. However, it remains unclear whether the old/new effects are sensitive to the nature of different semantic relations. The current ERP (event-related potentials) study aimed to address this, where picture pairs of thematic, taxonomic, and unrelated relations served as stimuli and participants were required to discriminate the pair type: intact, rearranged, “old + new”, or new. We confirmed both FN400 and LPC. Our findings, by comparing the occurrence and the amplitudes of these two components, implicate that the neural activity of associative recognition is sensitive to the semantic relation of stimuli and depends more on stimulus properties, that the familiarity of a single item can impact the neural activities in discriminating associative pairs, and that the interval length between encoding and test modulates the familiarity of unrelated pairs. In addition, the dissociation between FN400 and LPC reinforces the dual-process models.

## 1. Introduction

### 1.1. Theories for Item Memory and the Associated Old/New Effects

Thus far, single-process models and dual-process models are two widely mentioned theories discussing the memory of events (i.e., episodic memory that includes item memory and source memory, e.g., a word in red), where item memory mainly concerns the memory for the central details about the events and allow us to differentiate experienced items from novel ones [1,2,3,4,5]. According to the former theory, item memory depends upon a simple dimension that reflects its trace strength in the brain [2].

Dual-process models claim that item memory hinges on two functionally distinct mnemonic processes: familiarity and recollection. Familiarity is an automatic and fast-acting process that involves a generic feeling that something/someone is previously encountered; the recollection, by contrast, is a deliberate and time-consuming process with autonomous consciousness for studied episodes, which enables one to reinstate explicit contextual details associated with past encounters [4,5,6,7,8,9]. With the accumulation of research, the dual-process models are more supported. It has also been found that item memory can be accomplished either through the involvement of both familiarity and recollection processes or solely through the familiarity process [1,3].

In addition to the behavioural perspective, the brain activities engaged in item memory are also probed, including investigations utilizing the high temporal resolution technique of ERP [9,10,11,12,13,14,15,16]. They all reveal that the brain activities of the familiarity and recollection processes are dissociated. The ERP investigations usually concentrate on the old/new effects. By definition, the effects refer to the waveform contrasts between those elicited by the accurately identified old stimuli versus those elicited by the correctly rejected novel ones [9,11,12,14,15,16,17].

So far, two typical old/new effects of different temporal and topographical distributions in item recognition research are rendered. The first effect, which distributes over the central-frontal region during the epoch of approximately 300–500 ms post-stimulus onset (usually largest around 400 ms), manifests waveforms evoked by the correctly rejected novel items that are more negative-going than those by the accurately discriminated old ones, and this effect is also named the *FN400* [11,14,15,17,18]. The second effect, which emerges late and is larger over the parietal scalp around the time window of 500–800 ms, commonly presents an enhanced positivity for the accurately identified studied stimuli, and it is also termed the *LPC* [11,17,18,19,20,21,22,23]. The FN400 and LPC are thought to be the signals corresponding to familiarity and recollection processes, respectively [11,12,13,17,18,19,20,21,22,23]. 

### 1.2. Associative Memory

In daily life, one may undergo numerous events, and some of them are bound together. Research on the memory of the combination of distinct events causes great attention, and such capability to remember events experienced together is referred to as *associative memory* [6,7,24,25,26,27]. In a typical associative recognition paradigm, several strings of pairs (e.g., A-B, C-D, and E-F) are studied, and in the following test, participants are instructed to determine whether a pair is *intact* (the same as the one in the preceding study phase, e.g., A-B), *rearranged* (both items in the pair are studied but are recombined, e.g., C-F), or *new* (both items are novel, e.g., G-H) [7,8,25,27,28,29,30,31]. Studies by this paradigm generally demonstrate that both intact and new pairs are much easier to identify than rearranged ones [8,28].

The goal of associative memory is different from item memory as the former not only depends upon the feeling of previous experiences of the two items within a pair but also hinges on the determination of whether the two originally occur simultaneously, and this is essential to guarantee the successful differentiation of an intact pair from a rearranged pair. Buchler et al. have suggested that the discrimination of intact and rearranged pairs primarily depends upon the recollection process; if only the familiarity process is involved, the identification may fail [32]. By this token, if one considers an intact pair as rearranged, an error occurs, and vice versa [28,33]. 

In addition, to further disentangle the subtle aspect of associative information, a fourth pair, named the “old + new”, is introduced, in which one item originated from a studied pair while the other item is unexperienced before (e.g., D-I) [8,31,34]. Extant investigations have demonstrated that the discrimination of “old + new” pairs is much easier than those of rearranged ones, but is more difficult than those of intact and new ones [8,31,32,34]. Thus, the “old + new” pairs can also be used to differentiate item memory from associative memory, since the discrimination between intact and rearranged pairs requires associative information that is more based on the recollection process, while the discrimination between “old + new” and new pairs relies more on the item information that is based on the familiarity process; the differentiation of “old + new” pairs from intact and rearranged pairs might depend on both familiarity and recollection processes [31,34,35]. Therefore, we would consider the “old + new” pairs to treat this problem.

### 1.3. Two Routes to Enhance the Involvement of the Familiarity Process in Associative Memory

It has been discovered that associative recognition can also be accomplished through the familiarity process. Mayes et al. found that if the two items in a pair have common features that can facilitate their representations to overlap, the familiarity process would play a key role in associative memory [36]. Similarly, the levels of the unitization framework provide evidence that proposes higher accuracy for pairs whose items are easily integrated (e.g., integrate “black” and “board” into “blackboard”) than those that cannot, and a benefit for integrated pairs (or the unitization) is the production of more involvement of familiarity process [28,37,38,39,40]. There are two channels to enhance the unitization between items—the top-down method by controlling the encoding tasks, and the bottom-up method via improving the semantic relations between items [35,41,42,43,44].

In addition to the behavioural perspective, the contribution of unitization in associative memory is illuminated by neural activities as well, which offers evidence for the involvement of familiarity. For instance, research considering the top-down method confirms the involvement of the familiarity process in associative recognition by the ERP technique [35,45,46,47]. Bader et al. found that the recognition of word pairs from sentences only evoked the typical LPC component that correlated with the recollection process; by contrast, if the words were integrated into a novel concept, an early LPC (old/new effect) indexing the concept-driven familiarity was recorded [45]. 

Similarly, Wiegand et al. encouraged participants to encode the words of a pair by unitization, and subsequent recognition tests detected reliable old/new effects: if the pairs were shown in the same order as in the study phase, an early LPC old/new effect and the typical LPC were revealed; the reordered case, however, evoked both the typical FN400 and the typical LPC components [46]. Lu et al. found the FN400 when the stimuli were unitized in encoding, but the temporal distribution was different between words and pictures—rather later in pictures than in words [35]. This suggests the pattern of familiarity during successful retrieval depends on the stimulus properties. 

The bottom-up method to improve the semantic relation between items also gains neural evidence, including both words and pictures [35,42,47,48]. For instance, the recognition for the compound words revealed both FN400 and LPC, while the unrelated words only recorded the LPC component [47]. The recognition of synonym pairs could elicit both FN400 and LPC, while the non-synonym pairs only had the LPC component [48]. The research for picture pairs with a high semantic relation triggered both FN400 and LPC, while the unrelated cases only found the involvement of LPC [41,42,43].

In addition, research simultaneously controlling encoding tasks and the semantic relation of items reveals the contribution of familiarity to associative memory as well. For instance, Rhodes and Donaldson set two encoding situations of associative imagination and item imagination to associated pairs (e.g., “traffic-jam”) and unassociated pairs (e.g., “violin-guitar”) [49]. The test found that the former pairs elicited both FN400 and LPC under both encoding situations, while the latter pairs also recorded both components under the associative imagination encoding situation but only the LPC in the item imagination case. These findings demonstrate that associative imagination could facilitate the enrollment of familiarity in associative recognition.

### 1.4. Subtypes of Semantic Relation and the Neural Evidence

In recent years, semantic relations are subdivided into three different types: thematic, taxonomic, and unrelated [21,33,50,51,52]. Thematic relations generally refers to the relation of different items that play different roles in the same scene, which depends on an individual’s experience and emphasizes the external relations between items. Thematic relation items should have interaction or functional complementarity. For example, “lamp” and “table” hold a spatial relation because the former is usually located on the latter, and “joke” and “laugh” possess a cause-and-effect relation. Taxonomic relations are generally similarity based, where the similarity is defined as the shared attributes and overlapped features across items, such as “potato” and “eggplant” or “pen” and “pencil.” Comparatively, the items that are neither thematically related nor in a taxonomic relation are considered unrelated [21,33,51,52,53]. 

Prior explorations concerning different psychological processes offer convincing evidence that thematic relations and taxonomic relations involve distinct neural activities, but most of them are in identification tasks [33,50,52,54,55]. The brain differentiation between taxonomic and thematic relations is also explored in associative memory [33]. The ERP research conducted by Kriukova et al. showed that both thematic and taxonomic pairs yielded a significant FN400, and the pairs of taxonomic relation also elicited a reliable LPC, indicating that the enrollment of the recollection process in associative recognition was susceptible to the status of semantic relation in words [33]. 

### 1.5. Issues to Explore and Our Current Hypotheses

Despite all of this, there are still many unsolved issues in the research on the neural mechanisms of associative recognition. First, to the best of our knowledge, only one study has explored the impact of different semantic relations (including both the thematic and taxonomic ones) on associative recognition in words [33], and no attention is distributed to other materials, such as pictures. A question worth exploring is whether the modulation of different semantic relations on the neural mechanisms of associative recognition is material specific. Second, no study to date has considered the neural activities of “old + new” pairs, as the previous literature either concerned only the intact case or else focused on both intact and rearranged pairs [43,47,48]. The similarities and differences among these pairs await exploration. Third, extant neural investigations in associative recognition are nearly all conducted in the range of long-term memory, although the interval between encoding and test was inconsistent across the publications [43,44,45,46,47,48,56]. It is unclear yet whether the neural patterns would vary when the interval is shortened, e.g., in the range of short-term memory.

Based on these unsolved issues, the current study intended to provide evidence for the sensitivity of associative memory to semantic relation in short-term memory, including both behavioural and neural perspectives. Towards this end, intact, rearranged, and “old + new” picture pairs of thematic relation, taxonomic relation, and the unrelated ones were considered, and the corresponding ERP old/new effects were examined. The stimulus presentations were controlled in a continuous recognition paradigm. In this paradigm, the display of studied and novel stimuli is interwoven, namely, the second emergence of an event can be put before that of the first occurrence of another event [57,58]. Thus, this paradigm could help us to manipulate the interval between the encoding and the test of a pair within a relatively short range.

Behaviorally, we anticipated both correct response proportions and response speeds were modulated by both semantic relation and pair type. For retrieval-relevant old/new effects, the following hypotheses were predicted. (a) The old/new effects of FN400 and LPC, both exhibiting that the waveforms elicited by the correctly identified picture pairs were more positive-going than those by the correctly rejected new ones, should be recorded. Thus, compelling evidence could be provided for the dual-process models by demonstrating the involvement of both the familiarity and the recollection processes in associative memory. (b) We expected enhanced FN400 and LPC for intact pairs would be recorded, followed by rearranged and “old + new” cases successively. (c) The effects would behave distinctly in response to the factor of semantic relation, where the FN400 component would be more activated in thematic and taxonomic relations than in unrelated cases because the two former relations could enhance the unitization between items and the LPC component would be elicited across all the three semantic relations, thereby reinforcing the levels of the unitization framework. (d) We also considered the susceptibility of the neural activities to the interaction of pair type by semantic relation, for which the difference waveforms of both old/new effects were analyzed and the patterns in the above (b) would manifest larger in thematic relations than in taxonomic relations, which was followed by the unrelated cases. 

## 2. Experimental Procedure

### 2.1. Participants

Twenty undergraduates and graduates (11 males and 9 females, 22.46 ± 5.04 years) were recruited for the current experiment in exchange for course credit or RMB 50 for volunteering. All were native Chinese speakers and had normal or corrected-to-normal visual acuity. They all were right-handed as measured via the Edinburgh Handedness Questionnaire and had a reliability of 0.87 in Chinese participants [59], which followed previous memory investigations [9,11,26]. None of them reported any history of neurological or psychiatric disorders and/or colour blindness. Before participating in the experiment, each participant signed informed consent and was ignorant of our objectives. All protocols followed the guidelines established in the Declaration of Helsinki and were approved by the Research Ethics Committee of our university. By the end of the experiment, all participants received gratitude and a brief explanation.

To ensure the sample size was appropriate, we conducted a sensitivity power analysis applying G*Power Software v3.1 [60]. For this, the repeated-measures analysis of variance (ANOVA) for the current variables (see details in Section 2.2, where the measurement number was 12 and the correlation between measurements was 0.5) revealed that to achieve a small-to-medium-sized effect (*f* = 0.21) under the standard criteria (two-tailed α = 0.05, 1-β = 0.80), the minimum sample size was 17. Therefore, our current sample size met the requirement.

### 2.2. Design

The experiment was a 3 (semantic relation: thematic vs. taxonomic vs. unrelated) × 4 (pair type: intact vs. rearranged vs. “old + new” vs. new) within-subject design, where every semantic relation case had the four pair types of intact, rearranged, “old + new”, and new.

### 2.3. Materials

**Figure 1:**
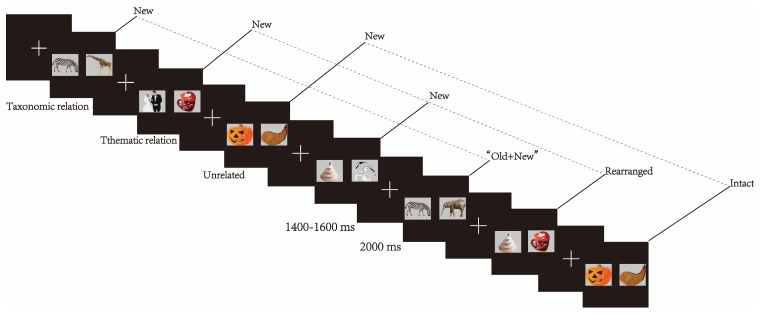
Schematic illustration of the experimental procedure with exemplars of picture pairs, illuminating the function of semantic relation by pair type. The procedure is designed via a continuous recognition paradigm. Participants are presented with a series of picture pairs and informed to differentiate among the four types of pairs: intact, rearranged, “old + new”, and new.

Before the formal experiment, we collected 1400 colourized pictures. Most of them were taken from the Hemera Photo-Objects Collection (of Canada), as in the previous literature [21,35,41,42], and a small amount were downloaded from the internet. Picture content included a variety of categories (e.g., people, animals, plants, landscapes, scenes, tools, and food), but they were not repeated, that is, we did not use pictures of different dogs or different pictures of one dog. The pictures were made consistent in size and brightness and displayed on a grey background by applying the Adobe Photoshop CC 2015 Software (see exemplars in Figure 1).

Before the formal experiment, two assessments were arranged to ensure the effectiveness of both thematic and taxonomic relations. For this, we first put the pictures into 700 pairs based on semantic classifications, in the same manner as Tibon and Levy [43] and Nie, Pan et al. [21]. Subsequently, we had 40 participants, who would not engage in the formal experiment, assess the semantic relations for the core contents of the two pictures geared to each pair on a five-point Likert scale, where “1” denoted highly unrelated and “5” signified extremely thematic in relation to the first assessment and extremely taxonomic in relation in the second assessment. Before the assessments, the meanings, along with exemplars, of both thematic and taxonomic relations were clearly described to participants to ensure the quality of the assessments.

Afterwards, the pairs whose scores were lower than 2.5 were classified as unrelated, and the pairs whose scores were larger than 3.5 in both assessments were eliminated. The elimination was mainly because thematic relation and taxonomic relation interweave sometimes. For instance, the pair of “bird” and “worm” can be classified as both thematic relation (because birds eat worms) and taxonomic relation (because both are animals). As a consequence, the ones selected as thematic and taxonomic relations all scored higher than 3.5, but with no repetition. Finally, we attained 546 picture pairs as experimental stimuli, 504 for the formal experiment, and the remaining 42 as practical trials.

The formal pairs were distributed across different semantic relations and pair types. To avoid interference across distinct pairs and reduce participants’ load, the total pool of the formal stimuli was assigned to six blocks, balanced on semantic relation and pair type. Each block had 144 pairs in total: 72 new (including 24 thematic relations, 24 taxonomic relations, and 24 unrelated), 24 intact, 24 rearranged, and 24 “old + new”. The last three pair types all had 8 pairs per semantic relation. 

The four pair types were differentiated in the following fashion. If the two pictures in a pair were both displayed first, the pair was new; if both were displayed the second time and were always paired together, the pair was intact; if a pair was recombined by the pictures belonging to two previously presented pairs, it was rearranged; if only one picture was redisplayed, the pair was “old + new”. For example, if the items of “pepper-tomato” and “radish-cabbage” were all displayed first, the pairs were new. Subsequently, when participants saw the pair of “pepper-tomato” again, it was intact, and the pair of “eggplant-cabbage” was rearranged, while “radish-cucumber” was “old + new”. All pairs were controlled on the semantic relation variable. That is, a picture should be always in one semantic relation (thematic or taxonomic or unrelated). Taking a rearranged pair as an example, the two pictures constituting it must be taken from two different pairs previously shown but should be in the same semantic relation constantly. This rule was also held for the “old + new” case.

### 2.4. Procedure

During the experiment, participants were comfortably seated in an electrically shielded room with dim lighting. Participants were instructed to engage in the practice trials plus six formal blocks and were instructed to fixate on the centre of the monitor and minimize eye blinks while pictures were displayed. The protocols (see descriptions below for details) were identical between the practice and the formal blocks. The practice trials were to familiarize participants with the experimental protocols.

In each block, task instructions were given first, which informed the participants to discriminate whether a pair was intact, rearranged, “old + new”, or new. If one pair was thought of as intact, the “←” key should be pressed and rearranged to press the “→” key; or if it was considered as “old + new” or new, the “↓” key should be pressed. The reason for setting one key for both “old + new” and new pairs was that our pilot experiment found that if one more key was required (i.e., one key for “old + new” pairs and another for new pairs), at least two major issues would result. The first was the decrease in correct response proportions that might cause the collapsed trials to not always be in line with the minimum requirement of the artefact-free trial for each condition per participant. The second was that the responses would be prolonged, triggering the response speeds to be far out of the latency windows of the old/new effects that we were concerned about. To overcome these two problems, we chose to have the same key for “old + new” and new pairs. Participants were also encouraged to respond as quickly and accurately as possible. The assignments of response keys and response fingers were counterbalanced among blocks and participants. 

Afterwards, trials of a white fixation cross and a picture pair were given. Within each trial, the fixation cross was first displayed in the centre of the computer monitor for 1400–1600 ms on a black background, followed by a pair lasting for 2000 ms. The two pictures of each pair were shown on the left and right sides of the fixation cross, ensuring a 4 cm blank distance between them. The displayed location of a represented picture was always the same as its first display. The next trial proceeded regardless of whether a response was made and the accuracy of the response. We applied the continuous recognition paradigm to display picture pairs, making sure there were 2–20 pairs (i.e., the interval was 7–70 s) between one new pair and its associated intact, rearranged, or “old + new” pair. Trials across different conditions emerged pseudo-randomly to ensure the pairs of the same semantic relation by pair type would not occur consecutively more than three times. 

The stimuli were programmed via Presentation 17.0 Software. Stimuli were displayed against a whole black background on a computer monitor. The monitor resolution was 1024 × 768 pixels and the refresh frequency was 100 Hz. The monitor was set 70 cm away from the participants. Pairs subtended a visual angle of 13.38° × 4.33°. To prevent interference across blocks and reduce participants’ load, a five-minute rest interval was always intermediated between two consecutive blocks. Figure 1 represents the schematic illustration of the experimental procedure with exemplars of picture pairs.

### 2.5. Electrophysiological Recording

Scalp electroencephalographic activity (EEG) was recorded continuously with Synamp amplifiers from 32 Ag/AgCl electrodes through the software and hardware of Neuroscan Company (Scan, SynAmps, Compumedics, El Paso, TX, USA). The electrodes were extended from the international 10/20 system. For the electrooculogram (EOG), two electrodes placed at the external canthi of both eyes measured the horizontal EOG, and two sites affixed on the supra- and infra-orbital ridges of the left eye were to record the vertical EOG. The EEG signals were amplified with a gain of 500 and digitized at a sampling rate of 500 Hz per channel. All channels were referenced to the right mastoid and were band-pass filtered from 0.05 to 40 Hz online, and scalp recordings were algebraically re-referenced to the average of both mastoids offline. All site impedances were maintained lower than 5 kΩ.

## 3. Data Analyses and Results

For both behavioural and ERP results, data were analyzed by the Software Package of IBM SPSS Statistics v22 (IBM Corporation, Amunk, NY, USA, 2014). ANOVAs were corrected using the Greenhouse-Geisser method when violating sphericity, while the degrees of freedom were the uncorrected ones. F ratios were reported with degrees of freedom and *p* values, and the Greenhouse-Geisser adjusted effect-size of partial eta-squared (η^2^). Whenever necessary, the Bonferroni correction was utilized to counteract the problem of multiple comparisons. All inferential analyses applied a two-tailed α = 0.05. Significant and concerning results were reported. The data of SPSS output could be found in Supplementary Materials.

### 3.1. Behavioral Analyses and the Data

**Figure 2:**
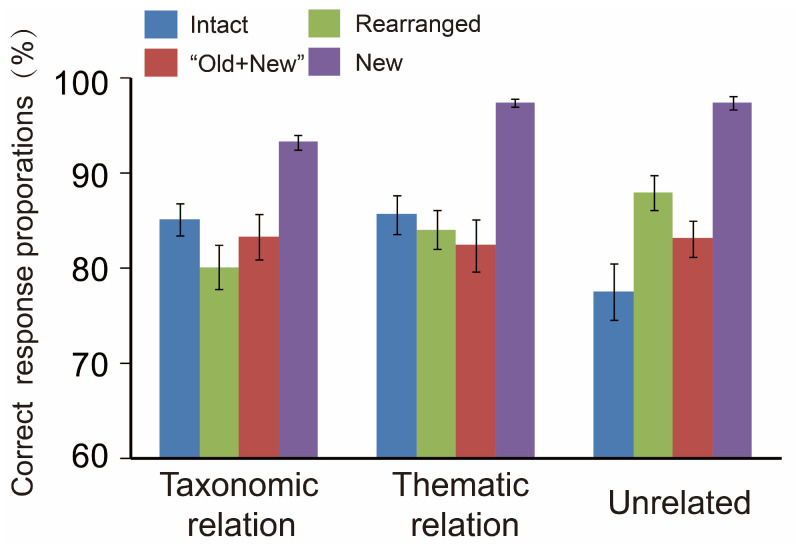
Correct response proportions as the function of pair type by semantic relation. Bars represent the standard errors.

**Figure 3:**
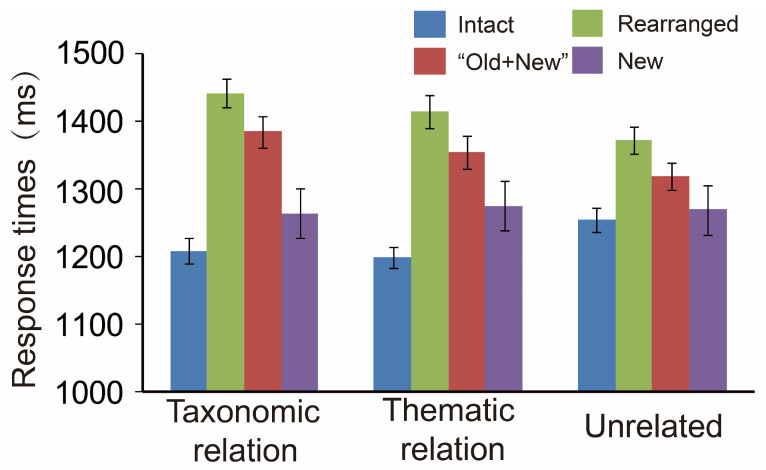
The response times (in ms) as the function of pair type by semantic relation. Bars represent the standard errors.

Figure 2 and Figure 3 illustrate the correct response proportions and the response times, respectively, and both are displayed as the function of semantic relation by pair type. To substantiate whether the behavioural data were susceptible to the current variables, the correct response proportions and response times were separately submitted to a 3 (semantic relation: thematic vs. taxonomic vs. unrelated) × 4 (pair type: intact vs. rearranged vs. “old + new” vs. new) repeated-measures ANOVA. 

Regarding the correct response proportions, the ANOVA did not reveal a significant main effect of semantic relation, *p* > 0.05, but a reliable main effect of pair type, *F* (3,57) = 30.09, *p* < 0.001, η^2^ = 0.613, and the two-way interaction, *F* (6,114) = 9.26, *p* < 0.001, η^2^ = 0.328. A simple effect test for the interaction demonstrated that the proportions of the thematic cases were modulated by pair type, that is, the one-way ANOVA for pair type (4 levels: intact vs. rearranged vs. “old + new” vs. new) in thematic cases showed *F* (2,38) = 34.59, *p* < 0.001, illuminating that new pairs performed superior to the other pair types, *ps* < 0.001. The proportions of the taxonomic situations were sensitive to pair type either, with the new ones holding much higher proportions than the other pairs, *ps* < 0.001; intact pairs behaved higher than rearranged ones, *p* = 0.006. Similarly, the unrelated cases also revealed the sensitivity to pair type, *F* (2,38) = 34.59, *p* < 0.001, with the new performing significantly better than the other pair types, *ps* < 0.001; the rearranged had much higher data than both intact and “old + new” pairs, *ps* ≤ 0.036; and intact pairs acted worse than the other pairs, *ps* ≤ 0.029. In sum, the above patterns demonstrate the interactive impact of semantic relation by pair type on correct response proportions. 

The same ANOVA for the response times revealed significant effects for both variables, *F* (2,38) = 5.85, *p* = 0.006, η^2^ = 0.236, and *F* (3,57) = 24.47, *p* < 0.001, η^2^ = 0.563; their interaction reached statistical conspicuousness either, *F* (6,114) = 12.83, *p* < 0.001, η^2^ = 0.403. The simple effect test for the interaction verified that the speed for all semantic relations was modulated by pair type. First, the one-way ANOVA for the variable of pair type (4 levels: intact vs. rearranged vs. “old + new” vs. new) found the speed in identifying thematic pairs differed as the function of pair type, *F* (2,38) = 32.72, *p* < 0.001, with intact pairs responding faster vs. rearranged and “old + new” ones, *ps* < 0.001; with rearranged ones behaving the slowest, *ps* < 0.001; and with “old + new” and new pairs sandwiched between them, although the “old + new” pairs had a longer speed than new ones, *p* < 0.001.

Second, the speed of taxonomic relations was also sensitive to pair type, *F* (2,38) = 15.12, *p* < 0.001, with intact pairs responding faster than both rearranged and “old + new” pairs, *ps* < 0.001; rearranged pairs holding a longer response than the rest ones, *ps* ≤ 0.033; and, with the intermediate pairs, new pairs responding faster than “old + new” ones, *p* = 0.002. Finally, the speed of unrelated pairs was susceptible to pair type as well, *F* (2,38) = 21.16, *p* < 0.001, with the intact pairs responding the fastest, the rearranged ones the slowest, and the other two intermediated between, *ps* ≤ 0.008. In brief, the response speed also acts as the function of semantic relation by pair type.

### 3.2. Electrophysiological Analyses and Results

EEG data were processed offline by the Scan 4.5 Software of Neuroscan Company (Scan, SynAmps, Compumedics, El Paso, TX, USA). Eye artefact correction was accomplished by applying a semi-automatic procedure before averaging, as in the extant literature [9,13,16,61]. Following correction, any trials that were contaminated by artefacts exceeding ± 100 µV were excluded before collapsing by employing a PCA-based algorithm used in the published literature [9,16,62]. The EEG was filtered with a band-pass from 0.05 to 40 Hz. Epochs of 1100 ms (including a 100 ms baseline and 1000 ms post-stimulus onset) were extracted from the continuous recording and corrected over the pre-stimulus interval of −100 ms. Through this, we acquired 12 grand-averaged waveforms, with each semantic relation containing the waveforms of intact, rearranged, “old + new”, and new pairs, all collapsed for the correctly identified trials. The minimum artefact-free trial number for each condition per participant was 16, conforming to the requirement described in the previous literature [9,11,12,16,63]. 

Based on previous ERP literature on recognition [13,16,21,64,65,66,67,68,69] and visual inspection on the current grand-averaged waveforms, two latency windows of 350–500 ms and 500–650 ms were extracted to measure FN400 and LPC, respectively. Four electrodes over the midline scalp regions, frontal (FZ), central (CZ), parietal (PZ), and occipital (OZ) regions, were selected. FN400 focused on the frontal region during 350–500 ms and LPC focused on the parietal region during 500–650 ms. To measure the old/new effects for each component of thematic relation, taxonomic relation, and unrelated conditions separately, the mean amplitudes of the correctly identified trials in each relation were delivered to a repeated-measures ANOVA of 2 (pair type: intact (or rearranged or “old + new”) vs. new) × 4 (scalp region: frontal vs. central vs. parietal vs. occipital). For this, 18 repeated-measures ANOVA were performed. Whenever there was a reliable interaction of pair type with scalp region, only the pair type sensitive data were reported. 

#### 3.2.1. ERP Results of Both FN400 and LPC

##### The Old/New Effects of Thematic Relation

**Figure 4:**
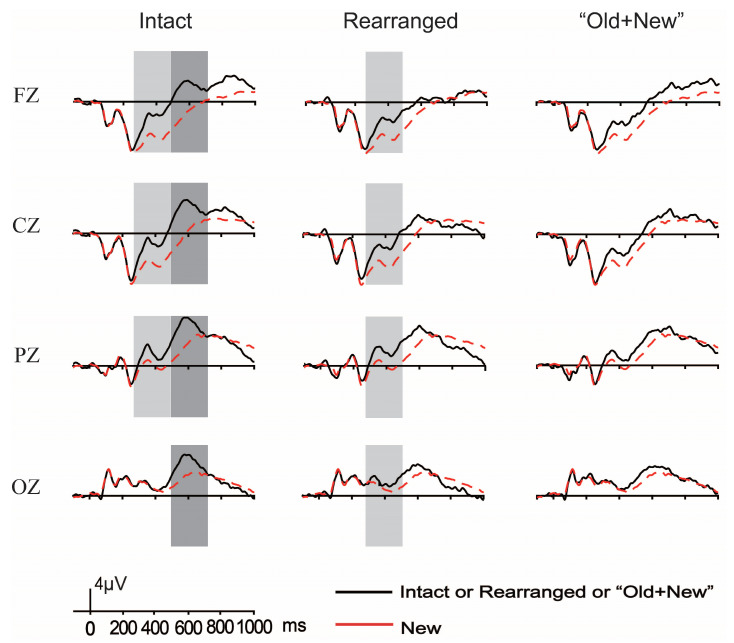
The grand-average ERP waveforms elicited by correctly rejected new pairs with those of correctly identified intact, rearranged, and “old + new” pairs of thematic relations, and the boxes mark the reliable FN400 and LPC.

**Figure 5:**
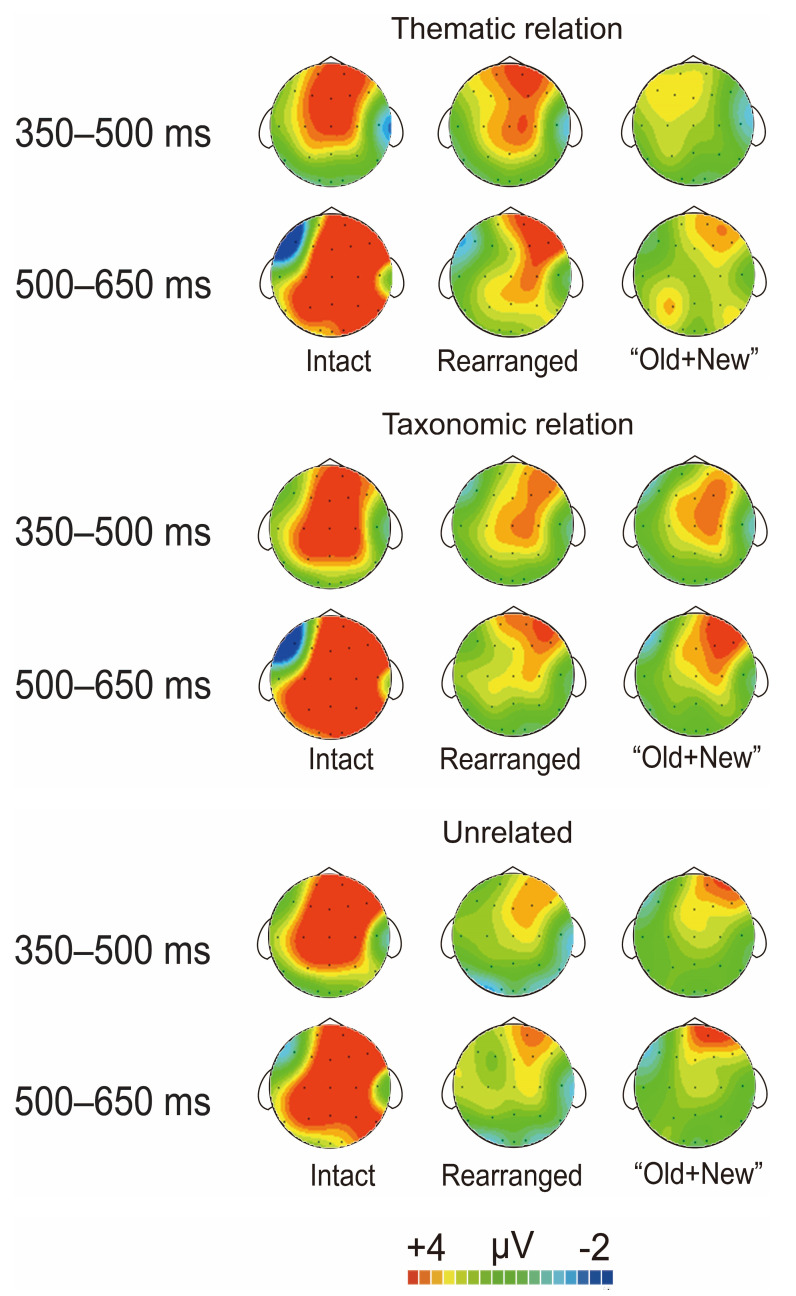
The topographical maps over the entire scalp extracted from difference waveforms between correctly rejected new pairs and correctly identified intact, rearranged, and “old + new” pairs, are shown within the latency windows of interest.

Figure 4 illustrates the grand-average ERP waveforms elicited by correctly rejected new pairs with those of correctly identified intact, rearranged, and “old + new” pairs under the thematic relation condition. Figure 5 plots the topographical maps over the entire scalp extracted from the difference waveforms between the correctly rejected new pairs and the correctly identified intact, rearranged, and “old + new” pairs, shown within the latency windows of interest.

FN400. For the time window of 350–500 ms, the repeated-measures ANOVA of pair type (intact vs. new) by scalp region (frontal vs. central vs. parietal vs. occipital) confirmed a reliable main effect of pair type, *F* (1,19) = 40.53, *p* < 0.001, η^2^ = 0.681, illuminating that the intact trials elicited more positive-going waveforms than the new counterparts; and the two-way interaction was significant, *F* (3,57) = 18.39, *p* < 0.001, η^2^ = 0.492. A subsidiary independent *t*-test for the pair type (intact vs. new) variable demonstrated the positivity exhibited over the frontal, central, and parietal regions, *ps* < 0.001. For the rearranged case, the main effect of pair type also reached statistical significance, *F* (1,19) = 40.28, *p* < 0.001, η^2^ = 0.679, revealing the amplitudes elicited by rearranged pairs were more positive-going compared with those by new ones. Pair type also interacted with scalp region, *F* (3,57) = 7.59, *p* = 0.003, η^2^ = 0.285. A subsidiary independent *t*-test of the pair type (rearranged vs. new) variable found that the difference between rearranged and new pairs was distributed over all the analyzed regions, *ps* ≤ 0.015. Next, positivity was elicited by “old + new” pairs than by new pairs, too, as there was a significant main effect of pair type, *F* (1,19) = 25.32, *p* < 0.001, η^2^ = 0.571, and an interaction of pair type by scalp region. A follow-up simple effect test for the pair type (“old + new” vs. new) variable confirmed that the positivity was significant across frontal, central, and parietal regions, *ps* < 0.001. In conclusion, regarding thematic relation, the three pair types of intact, rearranged, and “old + new” all record reliable FN400 over the frontal region.

LPC. Within the latency window of 500–650 ms, the repeated-measures ANOVA of pair type (intact vs. new) by scalp region (frontal vs. central vs. parietal vs. occipital) confirmed a reliable main effect of pair type, *F* (1,19) = 35.09, *p* < 0.001, η^2^ = 0.649, which showed that the amplitudes triggered by intact pairs were more positive-going than those by new ones. Pair type interacted with scalp region, *F* (3,57) = 5.79, *p* = 0.009, η^2^ = 0.234. A subsequent independent *t*-test of the pair type (intact vs. new) variable discovered the amplitude difference distributed over all four analyzed scalp regions, *ps* < 0.001. The repeated-measures ANOVA of pair type (rearranged vs. new) by scalp region (frontal vs. central vs. parietal vs. occipital) only attained the main effect of pair type, *F* (1,19) = 20.55, *p* < 0.001, η^2^ = 0.52, confirming that rearranged pairs held more positive waveforms than new ones. There was also a significant main effect of pair type that the “old + new” pairs behaved more positive-going in amplitudes than new ones, *F* (1,19) = 9.76, *p* = 0.006, η^2^ = 0.339. Such difference was also similar among all of the analyzed scalp regions, since the two variables did not interact with each other, *p* > 0.05. To sum up, we recorded reliable LPC for intact, rearranged, and “old + new” pairs in thematic relation.

##### The Old/New Effects of Taxonomic Relation

**Figure 6:**
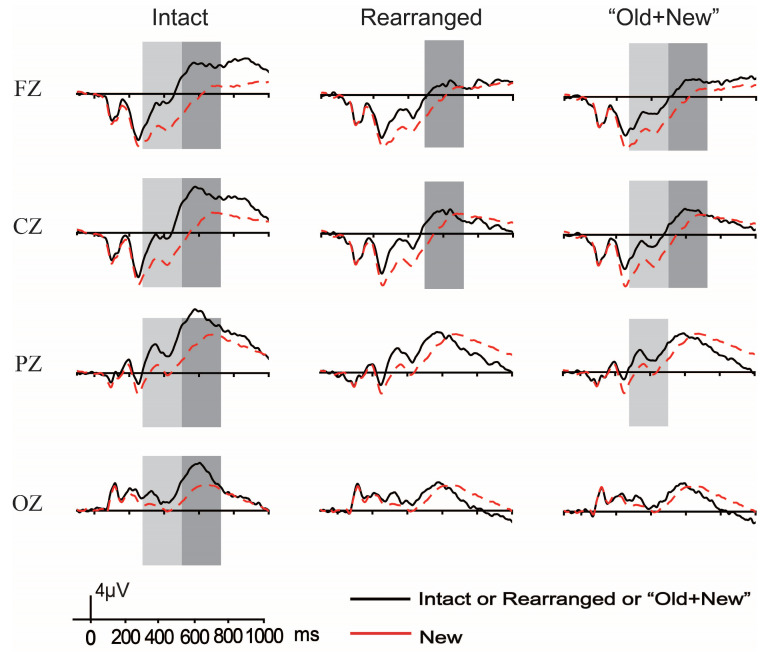
The grand-average ERP waveforms elicited by correctly rejected new pairs with those of correctly identified intact, rearranged, and “old + new” pairs of taxonomic relations, and the boxes mark the reliable FN400 and LPC.

Figure 6 illustrates the grand-average ERP waveforms elicited by correctly rejected new pairs with those of correctly identified intact, rearranged, and “old + new” pairs under the taxonomic relation condition; the corresponding topographical maps are plotted in Figure 5.

FN400. As for the time interval of 350–500 ms of taxonomic relation, we first made the repeated-measures ANOVA of pair type (intact vs. new) by scalp region (frontal vs. central vs. parietal vs. occipital), which found a significant main effect of pair type, *F* (1,19) = 75.20, *p* < 0.001, η^2^ = 0.798, with intact pairs activating more positive-going waveforms compared with new pairs. Furthermore, pair type interacted with the scalp region, *F* (3,57) = 23.29, *p* < 0.001, η^2^ = 0.551. The simple effect analysis by the independent *t*-test for the pair type (intact vs. new) variable proclaimed that the positivity for intact pairs was distributed over all of the concerned scalp regions, *ps* ≤ 0.003. As for the rearranged case, the repeated-measures ANOVA did not reveal a significant main effect of pair type and also its interaction with scalp region, *ps* > 0.05, demonstrating that the positivity was unmodulated by scalp region. When it turned to the “old + new” situation, the repeated-measures ANOVA showed both the main effect of pair type and the two-way interaction, *F* (1,19) = 15.01, *p* < 0.001, η^2^ = 0.441 and *F* (3,57) = 27.27, *p* < 0.001, η^2^ = 0.589. The follow-up simple effect test through the independent t-test for the variable of pair type (“old + new” vs. new) demonstrated the positive-going amplitudes for “old + new” pairs over frontal, central, and parietal regions, *ps* ≤ 0.005. These findings suggest that the taxonomic relation verifies the FN400 component for intact, rearranged, and “old + new” pairs. 

LPC. The LPC under the taxonomic relation focused on the epoch of 500–650 ms. We performed the repeated-measures ANOVA of pair type (intact (or rearranged or “old + new”) vs. new) by scalp region (frontal vs. central vs. parietal vs. occipital). The ANOVA for intact pairs confirmed the marked main effect of pair type, *F* (1,19) = 74.68, *p* < 0.001, η^2^ = 0.797, and also the two-way interaction, *F* (3,57) = 4.15, *p* = 0.026, η^2^ = 0.179. The simple effect test through the independent *t*-test of the pair type (intact vs. new) variable found that intact pairs triggered much larger positivity vs. new pairs across all the analyzed scalp regions, *ps* < 0.001. The rearranged pairs also recorded the pronounced main effect of pair type and its interaction with scalp region, *F* (1,19) = 8.58, *p* = 0.009, η^2^ = 0.311, and *F* (3,57) = 3.60, *p* = 0.033, η^2^ = 0.159. A subsidiary simple effect test by using the independent *t*-test for the pair type (rearranged vs. new) variable showed more positive amplitudes for rearranged pairs than new ones at both frontal and central regions, *ps* ≤ 0.02. Similarly, the ANOVA for the “old + new” case recorded the main effect of pair type and the two-way interaction, *F* (1,19) = 9.81, *p* = 0.005, η^2^ = 0.341 and *F* (3,57) = 9.16, *p* < 0.001, η^2^ = 0.325. Subsequent simple effect tests demonstrated that the “old + new” pairs had more positive-going waveforms than the new ones at both frontal and central regions, *ps* ≤ 0.019. Thus, we only have reliable LPC over the parietal region for intact pairs. 

##### The Old/New Effects of the Unrelated Condition

**Figure 7:**
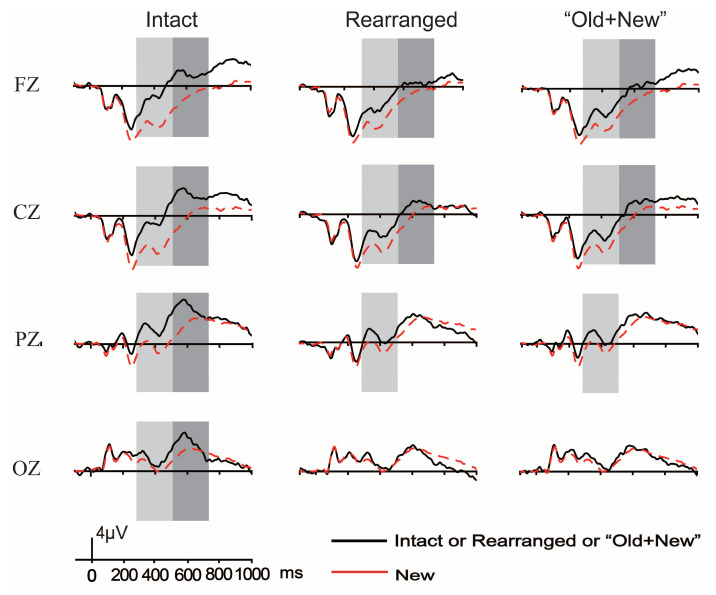
The grand-average ERP waveforms elicited by correctly rejected new pairs with those of correctly identified intact, rearranged, and “old + new” pairs under unrelated conditions, and the boxes mark the reliable FN400 and LPC.

Figure 7 illustrates the grand-average ERP waveforms elicited by correctly rejected new pairs with those of correctly identified intact, rearranged, and “old + new” pairs under unrelated conditions. The corresponding topographical maps are shown in Figure 5. For both FN400 and LPC, we performed three repeated-measures ANOVA of pair type (intact (or rearranged or “old + new”) vs. new) by scalp region (frontal vs. central vs. parietal vs. occipital).

FN400. We considered the latency window of 350–500 ms to measure the significance of FN400. Intact pairs had the marked main effect of pair type and the interaction of pair type by scalp region, *F* (1,19) = 52.69, *p* < 0.001, η^2^ = 0.735 and *F* (3,57) = 23.73, *p* < 0.001, η^2^ = 0.555. The simple effect test via the independent *t*-test for the variable of pair type (intact vs. new) showed that intact pairs elicited more positive-going amplitudes than new ones over all four concerned regions, *ps* ≤ 0.027. The repeated-measures ANOVA for the rearranged case found the main effect of pair type and the two-way interaction as well, *F* (1,19) = 11.29, *p* = 0.003, η^2^ = 0.373 and *F* (3,57) = 17.72, *p* < 0.001, η^2^ = 0.483. A subsidiary simple effect test for the pair type (rearranged vs. new) variable showed that there were more positive-going waveforms for rearranged vs. new pairs, and such difference emerged over the frontal, central, and parietal regions, *ps* ≤ 0.025. Next, the repeated-measures ANOVA for the “old + new” case had a significant main effect of pair type along with its interaction with scalp region, *F* (1,19) = 8.49, *p* = 0.009, η^2^ = 0.309 and *F* (3,57) = 10.88, *p* < 0.001, η^2^ = 0.364. The follow-up simple effect test for the pair type (“old + new” vs. new) variable revealed that “old + new” pairs triggered more positivity than new pairs that were distributed over the frontal, central, and parietal regions, *ps* ≤ 0.043. These findings suggest that we have reliable FN400 for intact, rearranged, and “old + new” pairs at the frontal region in unrelated situations. 

LPC. We considered the 500–650 ms interval to measure the significance of LPC, and the repeated-measures ANOVA for the intact case found the reliable main effect of pair type and the two-way interaction, *F* (1,19) = 27.21, *p* < 0.001, η^2^ = 0.589 and *F* (3,57) = 5.83, *p* = 0.011, η^2^ = 0.235. The subsequent simple effect test for the variable of pair type (intact vs. new) showed the amplitudes were more positive for intact pairs compared with new pairs at all interested regions, *ps* ≤ 0.025. Considering the rearranged situation, there was also the main effect of pair type and the two-way interaction, *F* (1,19) = 6.47, *p* = 0.020, η^2^ = 0.254 and *F* (3,57) = 7.43, *p* = 0.005, η^2^ = 0.281. Further simple effect tests for the pair type (rearranged vs. new) demonstrated the positivity at both frontal and central regions for rearranged pairs, *ps* ≤ 0.002. Next, the repeated-measures ANOVA for the “old + new” situation revealed the main effect of pair type along with its interaction with scalp region, *F* (1,19) = 7.33, *p* = 0.014, η^2^ = 0.278 and *F* (3,57) = 6.72, *p* = 0.006, η^2^ = 0.261. The follow-up simple effect test for the pair type (“old + new” vs. new) verified larger positivity in waveforms at both frontal and central regions for “old + new” pairs than new pairs, *ps* ≤ 0.002. Together, the unrelated circumstance only has the LPC effect for intact pairs.

#### 3.2.2. Analyses of the Difference Waveforms and Results

Since all three semantic relation circumstances recorded significant FN400 and LPC in different pair types, we further examined the contribution of pair type and semantic relation towards them. For this, the difference waveforms were extracted by subtracting the waveforms of new trials from those of intact, rearranged, and “old + new” ones per semantic relation. Similar to the aforesaid old/new effects, the latency windows of 350–500 ms and 500–650 ms were used to measure the difference waveforms of FN400 and LPC accordingly. Towards these ends, the following were our analyses from two different perspectives. 

##### Analyses of the Difference Waveforms per Semantic Relation and Results

To detect whether the difference waveforms of each semantic relation circumstance were modulated by the factors of pair type and scalp region, the difference waveforms, under each semantic relation, were delivered to a repeated-measures ANOVA of 3 (pair type: intact vs. rearranged vs. “old + new”) × 4 (scalp region: frontal vs. central vs. parietal vs. occipital), which was separately conducted for FN400 and LPC.

FN400. For the difference waveforms of FN400, the repeated-measures ANOVA for the thematic relation found there was a reliable main effect of pair type and the two-way interaction, *F* (2,38) = 3.34, *p* = 0.046, η^2^ = 0.149 and *F* (6,114) = 5.08, *p* = 0.003, η^2^ = 0.211. Subsequent simple effect examination of the pair type (3 levels: intact vs. rearranged vs. “old + new”) variable found that intact pairs elicited much greater difference waveforms than “old + new” pairs over the frontal region, *p* = 0.028; in the central region, the difference waveforms were much greater for both intact and rearranged pairs than “old + new” pairs, *ps* ≤ 0.050. Those over parietal and occipital regions did not observe any difference among the three pair types, *ps ≥* 0.246. Next, for taxonomic relation, the two-way repeated-measures ANOVA discovered that the main effect of pair type and its interaction with scalp region were both insignificant, *ps >* 0.05, suggesting that there was not any difference for taxonomic relation. The ANOVA for unrelated situations confirmed both the main effect of pair type and the two-way interaction, *F* (2,38) = 8.06, *p* = 0.003, η^2^ = 0.298, and *F* (6,114) = 4.40, *p* = 0.012, η^2^ = 0.188. The simple effect test by using the independent *t*-test for the pair type (intact vs. rearranged vs. “old + new”) variable found that in the frontal region, the difference waveforms were much greater for both intact and rearranged pairs than “old + new” pairs, *ps* ≤ 0.023; over the central and parietal regions, intact pairs had larger difference waveforms vs. both rearranged and “old + new” pairs, *ps* ≤ 0.012; and the difference waveforms at occipital region were insusceptible to pair type, *ps ≥* 0.749. Together, the difference waveforms of FN400 are sensitive to the interaction of pair type by scalp region, but this only holds in thematic relations and unrelated situations.

LPC. Considering the LPC, the mean difference waveforms were submitted to the repeated-measures ANOVA separately for the three semantic relation cases. For thematic relation, both the main effect of pair type and the two-way interaction was revealed, *F* (2,38) = 8.67, *p* < 0.001, η^2^ = 0.313, and *F* (6,114) = 3.781, *p* = 0.019, η^2^ = 0.166. Subsidiary simple effect analyses for the pair type (intact vs. rearranged vs. “old + new”) variable found that the difference waveforms all exhibited a pattern of intact pairs larger than rearranged and “old + new” pairs, and the pattern emerged over all four scalp regions, *ps* ≤ 0.028. The taxonomic relation had the main effect of pair type, *F* (2,38) = 13.68, *p* < 0.001, η^2^ = 0.419, but not the two-way interaction, *p* > 0.05. Subsequent multiple comparisons for the main effect verified that intact pairs had much higher difference waveforms than the other pairs, *ps* < 0.001. The unrelated condition also had the main effect of pair type, *F* (2,38) = 7.46, *p* = 0.005, η^2^ = 0.282, but not the two-way interaction, *p* > 0.05. Multiple comparisons for the main effect verified much higher difference waveforms for intact pairs than the other pairs, *ps* < 0.001. To sum up, the difference waveforms of LPC are modulated by pair type for all semantic relations.

##### Analyses of the Difference Waveforms per Pair Type and Results

To figure out whether the difference waveforms of each pair type were modulated by the factors of semantic relation and scalp region, the mean difference waveforms for each pair were submitted to a repeated-measures ANOVA of 3 (semantic relation: thematic vs. taxonomic vs. unrelated) × 4 (scalp region: frontal vs. central vs. parietal vs. occipital), which was separately conducted for FN400 and LPC.

FN400. During the latency window of FN400, the ANOVA for the intact pairs found that both the main effect of semantic relation and its interaction with the scalp region was insignificant, *ps >* 0.05. For the rearranged pairs, a similar insignificance was revealed, *ps >* 0.05. The ANOVA for the “old + new” pairs also did not confirm any significance, *ps >* 0.05. In sum, these data consistently demonstrate that the difference waveforms of FN400 are not modulated by the semantic relation of intact, rearranged, and “old + new” pairs.

LPC. Within the epoch of 500–650 ms, the ANOVA for intact pairs found that both the main effect of semantic relation and its interaction with scalp region was insignificant *ps >* 0.05. The rearranged pairs also did not reveal the significance, *ps >* 0.05. The “old + new” pairs also did not reveal the significance, *ps >* 0.05. In brief, we do not reveal the contribution of semantic relation in eliciting the difference waveforms of LPC for intact, rearranged, and “old + new” pairs.

### 3.3. Results of the Correlation between Accuracy and Old/New Effects

In addition to the reliability of behavioral and neural perspectives, we conducted further correlation analyses between the correct response proportions and the amplitudes of both FN400 and LPC. The results found that except for the intact pairs of the unrelated condition (which found a significant correlation of *r* = 0.539, *p* = 0.014), the data of the rest conditions did not show any correlation—all *ps* > 0.05. This indicates that the amplitudes of the current old/new effect are almost insensitive to the behavioural pattern in accuracy for thematic relation and taxonomic relation, and both include intact, rearranged, and “old + new” pairs.

## 4. Discussion

To probe whether the old/new effects are sensitive to the semantic relation of stimuli in associative memory of short-term range, we tested picture pairs of thematic, taxonomic, and unrelated relations in the forms of intact, rearranged, “old + new”, and new. Our data revealed both FN400 and LPC, while the patterns of these two effects were impacted by the manipulations of semantic relation, stimulus properties, old and new traits of association, and the interval length between encoding and test. In the following sections, we discuss the implications of the findings.

### 4.1. The Neural Activity of Associative Recognition Is Sensitive to the Semantic Relation of Stimuli and Depends More on Stimulus Properties

Our current study found reliable FN400 and LPC components, but the components showed different patterns across pairs of different semantic relations, and the current pattern of old/new effects is different from those in previous studies in words. 

Previous studies consistently show that the recognition of picture pairs with high semantic relation requires the engagement of both familiarity and recollection processes. For instance, Lv et al. [56] confirmed that the associative recognition of picture pairs that had a strong degree of integration not only depended on the early familiarity process but also the late recollection process; Tibon and Levy recorded both FN400 and LPC in recognizing picture pairs with high semantic relation [43]. Our current study also found reliable FN400 and LPC components. First, these findings are highly consistent with the anticipation that when the items are in high semantic relation, the associative recognition of picture pairs can facilitate the involvement of the familiarity process; as a consequence, the associative recognition is underpinned by both familiarity and recollection processes of the dual-process models [1,3,4,5].

Second, the involvement of the recollection processes is modulated by the semantic relation of stimuli. Our data showed that the intact, rearranged, and “old + new” pairs of thematic, taxonomic, and unrelated relations elicited a reliable FN400 component, the intact, rearranged, “old + new” pairs of thematic relations evoked a reliable LPC component, and the intact pairs of taxonomic and unrelated relations triggered a significant LPC component. That is to say, although all our concerned cases elicited the FN400, the LPC acted differently: the thematic relations elicited a reliable LPC component across all concerned pair types, and only the intact pairs of taxonomic and unrelated relations triggered significant LPC. This indicates that the involvement of the recollection process is sensitive to the semantic relations of pictures in associative memory. 

Third, the current results are different from the patterns in the words of Kriukova et al. [33]. Kriukova et al. found that the thematic relation circumstance only recorded the FN400 component [33]. In the current picture study, the thematic relation elicited both FN400 and LPC for intact and rearranged pairs, and the taxonomic and unrelated circumstances also elicited FN400 for intact and rearranged pairs; the LPC was only significant in taxonomic and unrelated circumstances for intact pairs. These findings demonstrate that, besides the semantic relation, the neural activity of associative recognition also depends more on stimulus properties.

According to the levels of the unitization framework [39], the inconsistent neural activities between words and pictures might be because the unitization degree of the two types of materials is not completely the same, i.e., higher in words than in pictures, and this has been verified by one recent study [35]. Lu et al. demonstrated that the associative memory of words elicited an early FN400 (the one like our current typical FN400), while pictures recorded a later FN400 than the typical effect. This suggests that the unitization of words is relatively stronger than that of pictures, which makes the early familiarity process play a key role in identifying the intact and rearranged pairs of words. 

The word pairs and picture pairs in Lu et al. are all unrelated, too, and the unitization is controlled by the top-down method, which requires participants to either form a whole concept for the two words in a pair or image an interactive scene for them [35]. Thus, the top-down method of controlling encoding tasks to promote unitization can enhance the engagement of the early familiarity process in associative recognition. Several studies have confirmed this by showing that when the two words in a pair are integrated into a new concept through encoding, subsequent associative recognition can mainly rely on the familiarity process) [35,47]. By contrast, our current pictures hold different semantic relations, and there is not any explicit requirement (such as the top-down instructions) for participants to integrate the two pictures of a pair into a concept. 

Despite all this, Lu et al. [35] and our findings consistently demonstrate that the neural activities in the associative memory of pictures behave differently from those in words, and the former requires both familiarity and recollection processes, while the words tend to rely more on the familiarity process when the unitization is high enough. This conclusion is reasonable because pictures need two specific phases—object identification and word production [21,70]. So, our current participants have to identify the object in a picture and name it subsequently, and this may prolong the unitization of pictures and largely cause the involvement of the recollection process.

An alternative account for the divergence between words and pictures might be the manipulations in different experiments, which then lead to more involvement in the recollection process in the current pictures. To be specific, the interval length between encoding and test is long (i.e., beyond 5 min) in Kriukova et al. [33] but is short (i.e., 7–70 s) in the current case; also, in the study of Kriukova et al., the encoding and test are separated rather than the current one making the studied and novel pairs mixed through the continuous recognition paradigm. Thus, participants in the study of Kriukova et al. could keep the same strategy during encoding, i.e., to identify the nature of each pair by pressing the required key; however, the current intact, rearranged, “old + new”, and new pairs are displayed in a mixed fashion, and one has to transform the strategies ceaselessly.

### 4.2. The Familiarity of a Single Item can Impact the Neural Activities in Discriminating Associative Pairs

One of our interests is whether the “old + new” pairs could also record reliable old/new effects, and the answer is an absolute yes, but the patterns differ from those of intact and rearranged pairs.

As mentioned above, extant studies have either only explored the neural mechanisms in identifying intact pairs or simultaneously in intact and rearranged pairs [41,42,47,48]. No matter which pair type, intact or rearranged, both items in each are studied and may hold high familiarity in the test. The current study is the first attempt to consider the “old + new” pair, in which only one item is studied while the other item is new and the familiarity of such a pair can also be explored. The current results showed that, for all three types of semantic relations, there was a significant difference in waveform amplitudes between “old + new” and new pairs over frontal, central, and parietal regions within the latency windows of 350–500 ms, i.e., we recorded the FN400 for “old + new” pairs across thematic, taxonomic, and unrelated circumstances. 

One previous study has shown that associative recognition can record two subtypes of early old/new effects that reflect two subtypes of familiarity: the first is the FN400 component over the frontal area that reflects the familiarity process caused by the experiment and the other is the old/new effect at the parietal region that is thought to index the concept-driven familiarity (i.e., the early LPC component) [46]. Thus, within the epoch of 350–500 ms, the current waveform difference between new and “old + new” pairs in the frontal region pertains to the one that reflects the familiarity FN400, while that over the parietal region is the reflection of the concept-driven familiarity. This not only suggests that the familiarity of the “old + new” pairs is significantly distinct from that of the new ones but also that the familiarity of a single item can impact brain activities in discriminating association information.

One question that should be addressed is whether the degree of familiarity is similar among intact, rearranged, and “old + new” pairs. Our answer is no. That is, the familiarity in intact pairs is stronger than that in “old + new” pairs. The analyses revealed much larger difference waveforms of the FN400 component in the former pairs than in the latter pairs, especially for thematic and unrelated circumstances. In terms of rearranged pairs, the amplitude of the difference waveforms of FN400 was not always different from that of “old + new” pairs, as the reliable difference solely occurred in unrelated conditions. So, the amplitude of FN400 for rearranged items was larger in absolute score than for “old + new” pairs but did not reach statistical significance in certain circumstances. Thus, it would inevitably lead to the integration being less strong than that of intact pairs. Regardless, the conclusion that the familiarity of a single item can impact neural activities in associative memory is indisputable. 

### 4.3. The Interval Length between Encoding and Test Can Modulate the Familiarity of Unrelated Pairs

Our last interest is whether there would record reliable old/new effects when the interval between encoding and test is shortened, such as in short-term memory interval. Our finding showed significant old/new effects, but the effects were somewhat different from those in long-term interval situations.

A large number of studies have shown that the recognition of unrelated pairs is completed based on the recollection process, as they only activate the neural activities reflecting such a process, the reason being that unrelated pairs are difficult to integrate [41,42,47,48]. Similar to the above results, the current study records the LPC component reflecting the recollection process in the intact pairs of unrelated cases. One thing that needs to be pinpointed is that the current study finds that in addition to intact, rearranged, and “old + new” pairs of thematic and taxonomic relations, the intact, rearranged, and “old + new” pairs of the unrelated case all evoke a reliable FN400 component.

One possible account for the occurrence of FN400 in the unrelated situation is that it acts similarly to thematic and taxonomic relations to a certain degree, that is, the unrelated pairs could also be integrated and promote subsequent retrieval, where participants might force themselves to create a reasonable semantic relation between the two unrelated items in a pair, such as to image a situation including the two items or make a sentence for them. The reason is that when participants discover the semantic relations for the thematic and taxonomic cases, they might also try their best to establish a semantic relation by integrating the two items of other pairs, such traces could be stored in short-term memory and subsequently promote the enrollment of the familiarity process in identifying intact and rearranged pairs of unrelated pairs. Thus, the recognition of unrelated pairs does not solely depend on the recollection process and can also depend on the familiarity process. Furthermore, the analyses found that the difference waveforms of FN400 do not differ across the current three types of semantic relations. This demonstrates that semantic relation can neither prohibit the emergence of FN400 nor limit its strength and thus the susceptibility of the familiarity process to semantic relation is faintish.

An alternative explanation is the interval length between encoding and test. When making a comparison across studies, an important difference between previous research and the current study is the interval length between encoding and test. Several previous investigations usually arrange the tests within the long-term memory range. By contrast, the interval we applied is situated in the range of short-term memory. It turns out that the interval length between encoding and test bears an important role in eliciting the involvement of the familiarity process in associative recognition of unrelated pairs.

However, if a long interval is sandwiched, such traces would decay and the involvement of familiarity is weakened largely, pushing participants to appeal to the controlled process of recollection to make their decisions. Many previous investigations find that the unrelated pairs could largely trigger the late old/new effect but without the early old/new effect, which includes the studies in both words [47,48] and pictures [41,42]. Combining ours and these previous investigations, a conclusion can be drawn firmly: the interval length between encoding and test can modulate the familiarity of unrelated pairs. That is, the integration of unrelated items decays faster than those of thematic and taxonomic relations, making the involvement of the familiarity process in unrelated items conditional to a short-term interval and absent when the interval length turns into a long-term memory situation. The mechanisms behind the similarities and differences between the short-term range and the long-term range are needed in order to explore further.

## 5. Limitations and Future Directions

There are still some thoughtless places in the current study that leave room for improvement. The first limitation is that our current case is conducted via the bottom-up method to enhance the involvement of the familiarity process in associative memory, and a great curiosity would be whether the effects would act the same as in the current case if a top-down method is utilized. Within the top-down method, the encoding task would artificially facilitate participants to enhance the possibility of unitization between the items per pair [44,46]. Therefore, we strongly recommend a logical next step to concern the top-down variant in future investigations for stimuli of different semantic relations.

Second, we highly recommend the stimulus emotionality. Studies have found that the old/new effects are sensitive to the stimulus emotionality in both item memory and source memory [9,11]. Further, our recent investigations have found a negative impairment effect in associative memory [8,31], which manifests that the negative pairs (i.e., both items in a pair are negative in valence) have fewer correct response proportions than positive and neutral ones. This corresponds to the theory of spontaneous interactive imagery, which claims that, compared to positive and neutral pairs, the spontaneous interactive imagery is relatively poorer in negative pairs when forming a between-item association, resulting in a worse performance in negative cases [31,71]. To our best knowledge, no study has considered stimulus emotionality in exploring the neural activities for thematic and taxonomic pairs, rendering future work necessary to regard it as an independent variable.

Third, we applied the continuous recognition paradigm to shorten the interval between an encoding pair and its corresponding test pair. It is unknown yet whether the old/new effects or the brain activities would recur differently when the interval is prolonged. For future directions, investigations are expected to explore the issue by prolonging the interval. Moreover, we guess the properties of the experimental paradigm, the continuous recognition paradigm, might be a potential factor for the shorter latency windows of our current two old/new effects than those in many previous investigations (i.e., 300–500 ms for FN400 and 500–800 ms for LPC as in previous investigations) [72,73,74]. This is an issue awaiting thorough exploration.

Last, it is well known that our knowledge is accumulated by experience and increases with age, and the knowledge of semantic relation is no exception. The experience concerning thematic and taxonomic relations might be different among other age groups, children, and elder adults from the current younger adults, according to which both the behavioural patterns and the neural activities in associative memory might differ among different age groups. One study has found that older adults show a similar pattern of unitization to that of young adults in neural activities [47], but this study does not differentiate the semantic relation in detail. Future research may consider the cross-age group as one potential influencing factor in associative memory for stimuli of different semantic relations.

## 6. Conclusions

To sum up, the current study provides new insight into the old/new effects of associative memory in the short-term range and the contribution of semantic relations of stimuli. We reveal both FN400 and LPC and their dissociation reinforces the dual-process models. The current new implications are: (a) the neural activity of associative recognition is sensitive to the semantic relation of stimuli and depends more on stimulus properties, (b) the familiarity of a single item can impact the neural activities in discriminating associative pairs, and (c) the interval length between encoding and test can modulate the familiarity of unrelated pairs.

## Data Availability

The datasets generated and/or analyzed and the materials of the current study are shared.

## Supplementary Materials

The following supporting information can be downloaded at: https://www.mdpi.com/article/10.3390/brainsci13040553/s1, The SPSS output.
